# Optimum O_2_:CH_4_ Ratio Promotes the Synergy between Aerobic Methanotrophs and Denitrifiers to Enhance Nitrogen Removal

**DOI:** 10.3389/fmicb.2017.01112

**Published:** 2017-06-16

**Authors:** Jing Zhu, Xingkun Xu, Mengdong Yuan, Hanghang Wu, Zhuang Ma, Weixiang Wu

**Affiliations:** Institute of Environmental Science and Technology, Zhejiang UniversityHangzhou, China

**Keywords:** aerobic methane oxidation, denitrification, O_2_:CH_4_ ratio, intermediate accumulation, thermodynamics

## Abstract

The O_2_:CH_4_ ratio significantly effects nitrogen removal in mixed cultures where aerobic methane oxidation is coupled with denitrification (AME-D). The goal of this study was to investigate nitrogen removal of the AME-D process at four different O_2_:CH_4_ ratios [0, 0.05, 0.25, and 1 (v/v)]. In batch tests, the highest denitrifying activity was observed when the O_2_:CH_4_ ratio was 0.25. At this ratio, the methanotrophs produced sufficient carbon sources for denitrifiers and the oxygen level did not inhibit nitrite removal. The results indicated that the synergy between methanotrophs and denitrifiers was significantly improved, thereby achieving a greater capacity of nitrogen removal. Based on thermodynamic and chemical analyses, methanol, butyrate, and formaldehyde could be the main trophic links of AME-D process in our study. Our research provides valuable information for improving the practical application of the AME-D systems.

## Introduction

Biological denitrification, following nitrification, is widely used to remove nitrogen from wastewater. It includes four reduction steps: (1) from nitrate (${\text{NO}}_{3}^{-}$) to nitrite (${\text{NO}}_{2}^{-}$), (2) from ${\text{NO}}_{2}^{-}$ to nitric oxide (NO), (3) from NO to nitrous oxide (N_2_O) and (4) from N_2_O to dinitrogen (N_2_) (Zumft, 1997). Theoretically, ${\text{NO}}_{3}^{-}$ and ${\text{NO}}_{2}^{-}$ can be completely reduced to N_2_ in the presence of enough carbon sources if the microbes are equipped with full set of denitrification genes (Rittmann and McCarty, 2001). Among the four steps of denitrification, the reduction of soluble nitrite by nitrite reductase into gas is the key step and considered as the symbol of permanent removal of nitrogen from the aquatic ecosystem (Saunders and Kalff, 2001; Philippot and Hallin, 2005). Currently, carbons in the forms of methanol, ethanol and acetate are frequently supplemented for complete denitrification in wastewater treatment systems. However, the addition of external carbon sources inevitably increases the operational cost of wastewater treatment. Methane (CH_4_), a greenhouse gas, is readily available in many wastewater treatment plants and landfills and has a potential as an electron donor to replace traditional carbon sources for denitrification in nitrate-contaminated wastewater treatment (Modin et al., 2007). It was successfully demonstrated as a carbon source for denitrification in the presence of oxygen for the first time in 1978 (Rhee and Fuhs, 1978). This process was defined as aerobic methane oxidation coupled with denitrification (AME-D) (all the following discussions about this process are based on wastewater treatment systems). The AME-D process is a promising and realistic alternative to conventional biological treatment of nitrate-rich wastewaters. However, the nitrogen removal performance of AME-D systems is required to be higher than conventional systems for practical applications. Basic knowledge regarding operational parameters that affect the AME-D process is highly needed for the system process design.

**Figure 1:**
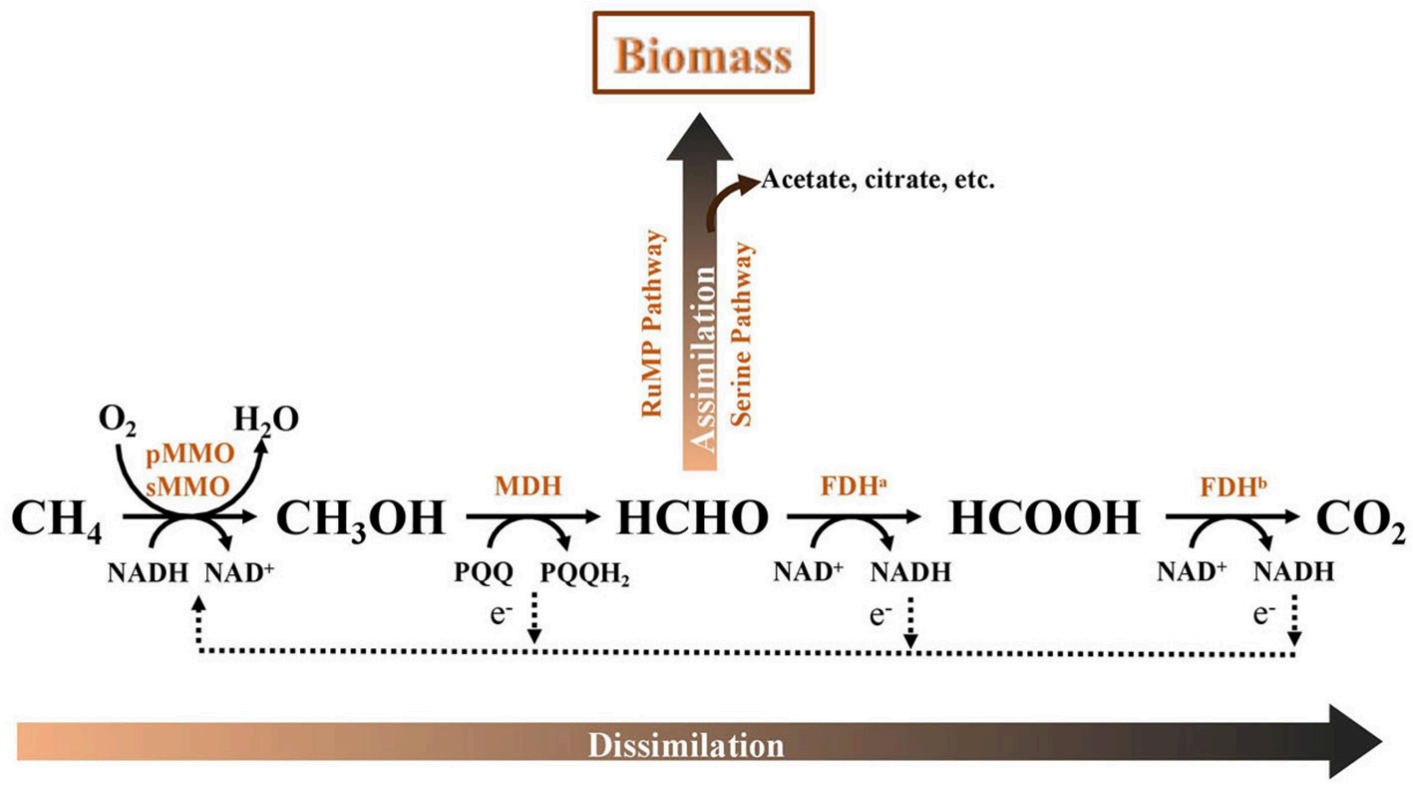
Traditional metabolism of methane in aerobic methanotrophs (modified from Zhu et al., 2016). pMMO, particulate methane monooxygenase; sMMO, soluble methane monooxygenase; MDH, methanol dehydrogenase; FDH^a^, formaldehyde dehydrogenase; FDH^b^, formate dehydrogenase; RuMP, ribulose monophosphate pathway. PQQ, pyrroloquinolinequinone; NAD^+^, oxidized form of nicotinamide-adenine dinucleotide; NADH, reduced form of nicotinamide-adenine dinucleotide.

The AME-D process is a synergistic collaboration between aerobic methanotrophs and denitrifying bacteria. Aerobic methanotrophs are a group of microorganisms capable of utilizing CH_4_ as a carbon and energy source. These microbes can oxidize CH_4_ to carbon dioxide (CO_2_) in the presence of oxygen (O_2_). Metabolic pathways of methane oxidation in aerobic methanotrophs are comprehensively summarized by other researchers (Trotsenko and Murrell, 2008; Zhu et al., 2016; Figure 1). Briefly, CH_4_ is initially catalyzed by methane monooxygenase (MMO), soluable MMO (sMMO) or particulate MMO (pMMO), to produce methanol as the first intermediate. Afterwards, methanol is transformed into formaldehyde by methanol dehydrogenase. Formaldehyde may be assimilated into biomass through the ribulose monophosphate pathway or the serine pathway, releasing multi-carbon intermediates such as acetate and citrate. Alternatively, formaldehyde can be dissimilated to CO_2_ via formate for energy production. It was demonstrated that dissolved organic intermediates, such as methanol (Meschner and Hamer, 1985), formaldehyde (Liu et al., 2014), acetate (Costa et al., 2000), which were released during aerobic methane oxidation, could be used as carbon sources for co-existing denitrifiers. On the metabolic pathways, denitrification in the AME-D process consists of two critical steps that may occur simultaneously or sequentially. The first is methane oxidation to release carbon sources, and the second is the use of these carbon compounds for denitrification (i.e., nitrate or nitrite removal). Therefore, operational parameters that affect the organic carbons excreted by methanotrophs for denitrification would have a critical impact on the nitrogen removal of AME-D process. Because of the well-known inhibition of excessive O_2_ on denitrification (Zumft, 1997), supply of enough electron donors and well-control of O_2_ level are two critical strategies to improve nitrogen removal and promote complete denitrification (${\text{NO}}_{3}^{-}$-${\text{NO}}_{2}^{-}$-NO-N_2_O-N_2_) in the AME-D systems.

It has been demonstrated that the O_2_:CH_4_ ratio is an essential parameter to regulate the carbon flow from CH_4_ to biomass and CO_2_, which impacts the production of carbon sources for nitrogen removal in the AME-D process. Morinaga et al. (1979) and Costa et al. (2000) discovered that methanotrophic strains could excrete formaldehyde and acetate, when the O_2_:CH_4_ ratio was lower than 1.0 (i.e., oxygen-limited). In contrast, no organic metabolites were detected when O_2_:CH_4_ ratio was higher than 1.0 (i.e., methane-limited). Kalyuzhnaya et al. (2013) further studied the pathway of intermediates production by aerobic methanotrophs under oxygen-limited conditions and discovered that acetate, lactate, and even hydrogen could be released during novel fermentation-related methanotrophy. Besides, oxygen is another critical factor to influence nitrogen removal in the AME-D process. Aerobic methanotrophs require enough O_2_ to utilize CH_4_, whereas excessive O_2_ will inhibit denitrifiers to reduce nitrogen (Modin et al., 2007). To date, environmental conditions that are favorable for both aerobic methanotrophs and denitrifiers are still unclear. Determination of an optimal O_2_:CH_4_ ratio which balances the requirements of carbon source and O_2_ could be an effective way for improving denitrifying performance in the AME-D process.

The aim of this research was to investigate the impact of the O_2_:CH_4_ ratio on nitrogen removal in the AME-D process and to determine the optimal ratio resulting in the highest nitrogen removal performance. It was hypothesized that intermediates released by methanotrophs primarily control nitrogen removal when O_2_:CH_4_ ratio is below the optimal value, whereas O_2_ level mainly controls nitrogen removal when O_2_:CH_4_ ratio is over the optimal. According to the stoichiometric (Equations 1 and 2) of the AME-D process (Zhu et al., 2016), the O_2_:CH_4_ ratio should be maintained below 1.1:1 (more specifically 0, 0.05, 0.25, and 1.0 in this study) to avoid excessive O_2_ inhibition on denitrification.

(1) $$\begin{array}{l} {\text{CH}}_{4}+1.1{\text{O}}_{2}+0.7{\text{2NO}}_{3}^{-}+0.72{\text{H}}^{+}=0.36{\text{N}}_{2}+{\text{CO}}_{2}\\ \;+\;2.36{\text{H}}_{2}\text{O} \end{array}$$

(2) $$\begin{array}{l} {\text{CH}}_{4}+1.1{\text{O}}_{2}+1.2{\text{NO}}_{2}^{-}+1.2{\text{H}}^{+}=0.6{\text{N}}_{2}+{\text{CO}}_{2}\\ \;+\;2.6{\text{H}}_{2}\text{O} \end{array}$$

## Materials and methods

### Sludge preparation

The sludge used in this study was collected from an AME-D culture that was enriched for more than 1 year in a batch bioreactor with continuous CH_4_ supply. The sludge was pre-incubated in a nitrite-containing medium under anoxic conditions in the dark at 25°C for 3 days to eliminate residual organic carbon sources. Then the sludge was centrifuged at 3,300 g for 5 min and the supernatant was discarded. The sludge pellet was resuspended and washed in sterile phosphate buffer solution (pH = 6.8) for three times to further remove extracellular organic carbons. Subsequently, it was centrifuged at higher speed of 14,000 g for 10 min to ensure complete precipitation of suspended cells. The experiments were initiated when no organic carbon was present in the supernatant. The organic carbon was determined with a Total Organic Carbon (TOC) analyzer (MultiN/C3100, Analytikjena, Germany). After all residual organics were removed, the sludge was re-suspended in the basal medium (Liu et al., 2014) at a mixed liquor suspended solid (MLSS) concentration of 17,735 mg/L. The basal medium contained the following components in 1 liter (L) distilled water: 1,250 mg KHCO_3_, 50 mg KH_2_PO_4_, 300 mg CaCl_2_·2H_2_O, 200 mg MgSO_4_·7H_2_O, 345 mg NaNO_2_, 1.0 mL acidic trace element solution and 1.0 mL alkaline trace element solution. Constituents of the acidic and alkaline trace element solution were described by Liu et al. (2014). Inorganic nitrogenous compounds were prepared from NaNO_2_ to result in a concentration of ${\text{NO}}_{2}^{-}$-N in the basal medium of 70 mg/L. Ammonium (${\text{NH}}_{4}^{+}$) and ${\text{NO}}_{3}^{-}$ were also supplied at low concentrations, 0.07 and 3.82 mg/L, respectively, to serve as nitrogen sources for microbes in the AME-D system. The pH of the medium was 7.2.

### Batch experiment

Batch experiment was conducted using 10 mL of the prepared sludge and 20 mL of the basal medium in a 150 mL glass vial. Freshly prepared mixture of the basal medium and the sludge was sparged with CH_4_ (99.99%) for 5 min. The vial was crimp-sealed with a butyl rubber stopper. Different volumes of CH_4_ (0, 6, 24, and 60 mL) were withdrawn from the headspace of the vials with a gas-lock syringe. Afterwards, the same volume of pure O_2_ (99.99%) was injected into the headspace of vials. The final compositions of O_2_ and CH_4_ in the gas mixtures of each treatment were shown in Table 1. Un-inoculated media was used as blank controls to test for leakage and non-biological chemical transformations. Triplicate samples were incubated at 28°C in shaking incubator at 180 rpm in the dark for 60 h. CH_4_ and nitrogen (${\text{NO}}_{3}^{-}$-N, ${\text{NO}}_{2}^{-}$-N and ${\text{NH}}_{4}^{+}$-N) consumptions and intermediate production were monitored in each treatment.

**Table 1:** Experimental set up for aerobic methane oxidation coupled with denitrification (AME-D) process.

**Treatment**	**Volume of the sludge (mL)**	**Volume of the deionized water (mL)**	**Volume of the basal medium (mL)**	**Volume of gas in the headspace**		**Percentage of gas in the headspace**	
				**O_2_ (mL)**	**CH_4_ (mL)**	**O_2_ (%)**	**CH_4_ (%)**
Treatment 1 (O_2_: CH_4_ = 0)	10	−	20	0	120	0	100
Un−inoculated control 1	−	10	20	0	120	0	100
Treatment 2 (O_2_: CH_4_ = 0.05)	10	−	20	6	114	5	95
Un−inoculated control 2	−	10	20	6	114	5	95
Treatment 3 (O_2_: CH_4_ = 0.25)	10	−	20	24	96	20	80
Un−inoculated control 3	−	10	20	24	96	20	80
Treatment 4 (O_2_: CH_4_ = 1.0)	10	−	20	60	60	50	50
Un−inoculated control 4	−	10	20	60	60	50	50

### Chemical analysis

The ${\text{NO}}_{3}^{-}$-N, ${\text{NO}}_{2}^{-}$-N, and ${\text{NH}}_{4}^{+}$-N concentrations were determined by ultraviolet spectrophotometry (UV-5300PC, METASH, China). The measurement of volatile suspended solid (VSS) was performed according to standard methods (American Public Health Association, American Water Works Association, Water Pollution Control Federation, 1989). In this study, nitrite removal expressed as mmol nitrite consumed per gram of biomass per day i.e., mmol ${\text{NO}}_{2}^{-}$-N/gVSS/d. To quantify CH_4_ content in the headspace of each vial, a sample of 0.5 mL was removed from the headspace of the vial with a gas-lock syringe. The sample was analyzed with a gas chromatograph (GC) equipped with a thermal conductivity detector under the conditions previously described by Zhang et al. (2014). Methane oxidation activity was expressed as CH_4_ consumed per gram of biomass per day i.e., mmol CH_4_/gVSS/d (Wang et al., 2008). NO concentration was measured by GC-mass spectrometry (6890N GC-5973MS, Agilent, USA) with the methods described by Leone et al. (1994). N_2_O concentration in the vial headspace was determined by a GC (GC-14B, Shimadzu, Japan) equipped with an electron capture detector and a Porapak Q column maintained at 330 K. Intermediate (methanol, formaldehyde, formate, acetate, and other potential organics) concentrations were determined with a high performance liquid chromatograph using the methods described by Thalasso et al. (1997).

### DNA extraction

Sludge samples were analyzed for DNA before and after incubation. Three independent DNA extractions of each treatment were performed from 30 mg of sludge using a FastDNA SPIN Kit for Soil (MP Biomedical, LLC, Ohio, USA). The extraction was performed according to the manufacturer's instructions. The concentrations and the quality of DNA samples were measured with a Nanodrop analyzer (Thermo Scientific, Wilmington, DE, USA). Extracted DNA was stored at −20°C prior to subsequent analyses.

### Quantification of functional genes

The abundance of aerobic methanotrophs and denitrifiers was estimated through quantitative PCR (q-PCR). Due to the low level of *mmoX*-harboring methanotrophs in the enrichment and high enough Cu concentration (2 μM) in the medium to inhibit the expression of *mmoX* gene (Takeguchi et al., 1997), the function of sMMO encoded by *mmoX* gene was considered to be negligible. Therefore, only *pmoA* gene encoding a subunit of pMMO was used to investigate the abundance variation of aerobic methanotrophs in this study. For denitrifiers, the *nirK* gene encoding copper nitrite reductase and the *nirS* gene encoding cytochrome cd_1_-containing nitrite reductase were used as the biomarkers. The quantification was based on the intensity of SYBR Green dye fluorescence, which can bind to double-stranded DNA. Standard curve for each gene were generated using a 10-fold dilution series of the linearized plasmid standard (10^−1^–10^−6^ ng) ranging from 10^8^ to 10^3^ copies. Each qPCR assay (25 μL) included 12.5 μL of 2 × SYBR Premix Ex Taq (Takara, Dalian, China), 1 μL of each forward and reverse primer (20 μM), either 1 μL of template DNA or the standard vector plasmid of the clones grown as single cellular suspension. The optimized thermal conditions and primers used for each gene can be viewed in the Supplementary Table 1. All real-time PCR assays were performed in triplicate for each sample in a Bio-Rad CFX1000 Thermal Cycler. All PCR runs included negative controls that did not contain DNA templates. The gene copy numbers were determined by comparing threshold cycles obtained in each PCR run with those of known standard DNA concentrations. Standard curves were obtained using serial dilutions of linearized plasmids containing cloned *pmoA, nirK*, and *nirS* genes.

### Statistical analysis

All data are presented as means and standard deviations. Analysis of variance and least significant difference (LSD) tests at the 5% level were used to determine the statistical significance of different treatments. Any differences with *p* ≥ 0.05 were not considered as statistically significant. The relationships between *nirK/nirS* gene copies and the nitrite removals at four treatments were tested with linear regression analyses using SPSS 20.0 for Windows (SPSS Inc., Chicago, IL).

## Results

### Methane oxidation activity and intermediates accumulation at different O_2_:CH_4_ ratios

**Figure 2:**
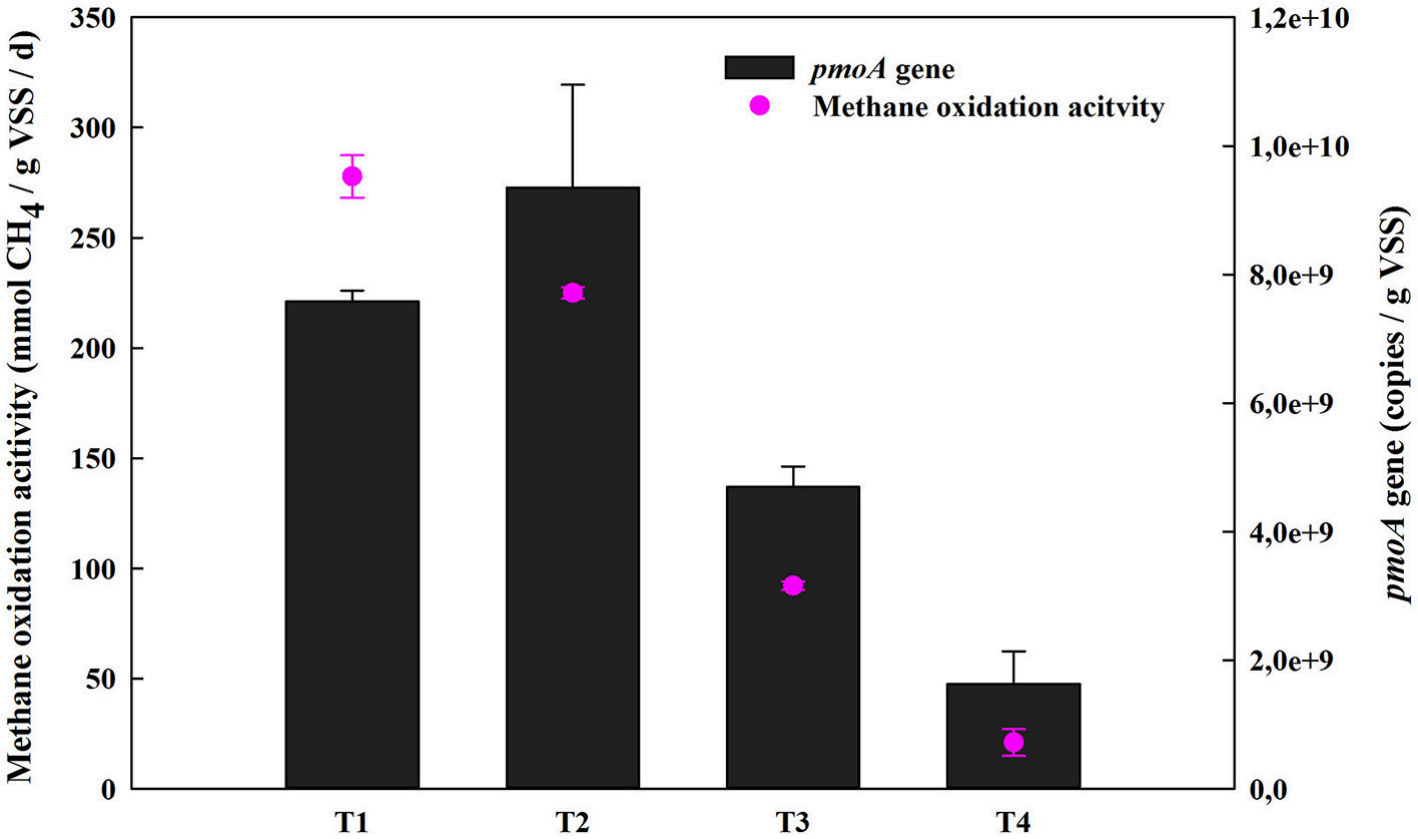
Methane oxidation activities of the consortia and the abundance of *pmoA* gene at different O_2_:CH_4_ ratios in aerobic methane oxidation coupled with denitrification (AME-D) process. T1, Treatment 1 (0% O_2_ and 100% CH_4_); T2, Treatment 2 (5% O_2_ and 95% CH_4_); T3, Treatment 3 (20% O_2_ and 80% CH_4_); T4, Treatment 4 (50% O_2_ and 50% CH_4_).

**Figure 3:**
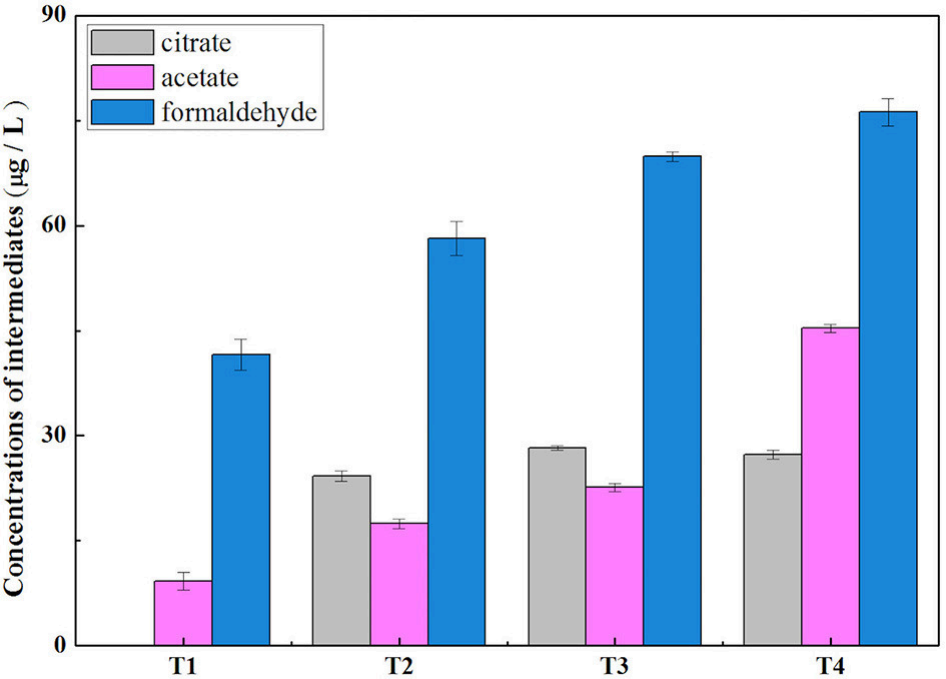
Accumulation of acetate, citrate and formaldehyde in the liquid at different O_2_:CH_4_ ratios in aerobic methane oxidation coupled with denitrification (AME-D) process. T1, Treatment 1 (0% O_2_ and 100% CH_4_); T2, Treatment 2 (5% O_2_ and 95% CH_4_); T3, Treatment 3 (20% O_2_ and 80% CH_4_); T4, Treatment 4 (50% O_2_ and 50% CH_4_).

Methane oxidation activity and concentrations of extracellular metabolites (methanol, formaldehyde, formate, and acetate, etc.) were determined under four different O_2_:CH_4_ ratios. As shown in Figure 2, methane oxidation activity substantially decreased from 277.80 mmol/gVSS/d to 21.06 mmol/gVSS/d when the O_2_:CH_4_ ratio was increased from 0 to 1 (*p* < 0.05). With the exception of treatment 1 at an O_2_:CH_4_ ratio of 0, a similar trend was observed for qPCR data of the *pmoA* gene. The *pmoA* gene abundance decreased almost an order of magnitude (from 9.36 × 10^9^ to 1.63 × 10^9^ copies per g dry biomass) when the O_2_:CH_4_ ratio was increased from 0.05 to 1 (Figure 2). The three primary metabolites observed in the bulk media were formaldehyde, acetate and citrate. Their concentrations went up from 42 to 76 μg/L (formaldehyde), 11 to 45 μg/L (acetate) and 0 to 28 μg/L (citrate), respectively, when O_2_:CH_4_ ratio was increased from 0 to 1 (Figure 3). Methanol, formate and butyrate were considered as three trace metabolites and their concentrations were lower than 1.20 μg/L (Supplementary Figure 1). However, methanol were not detected in the treatment 1 in the absence of O_2_.

### Nitrite removal at different O_2_:CH_4_ ratios

**Figure 4:**
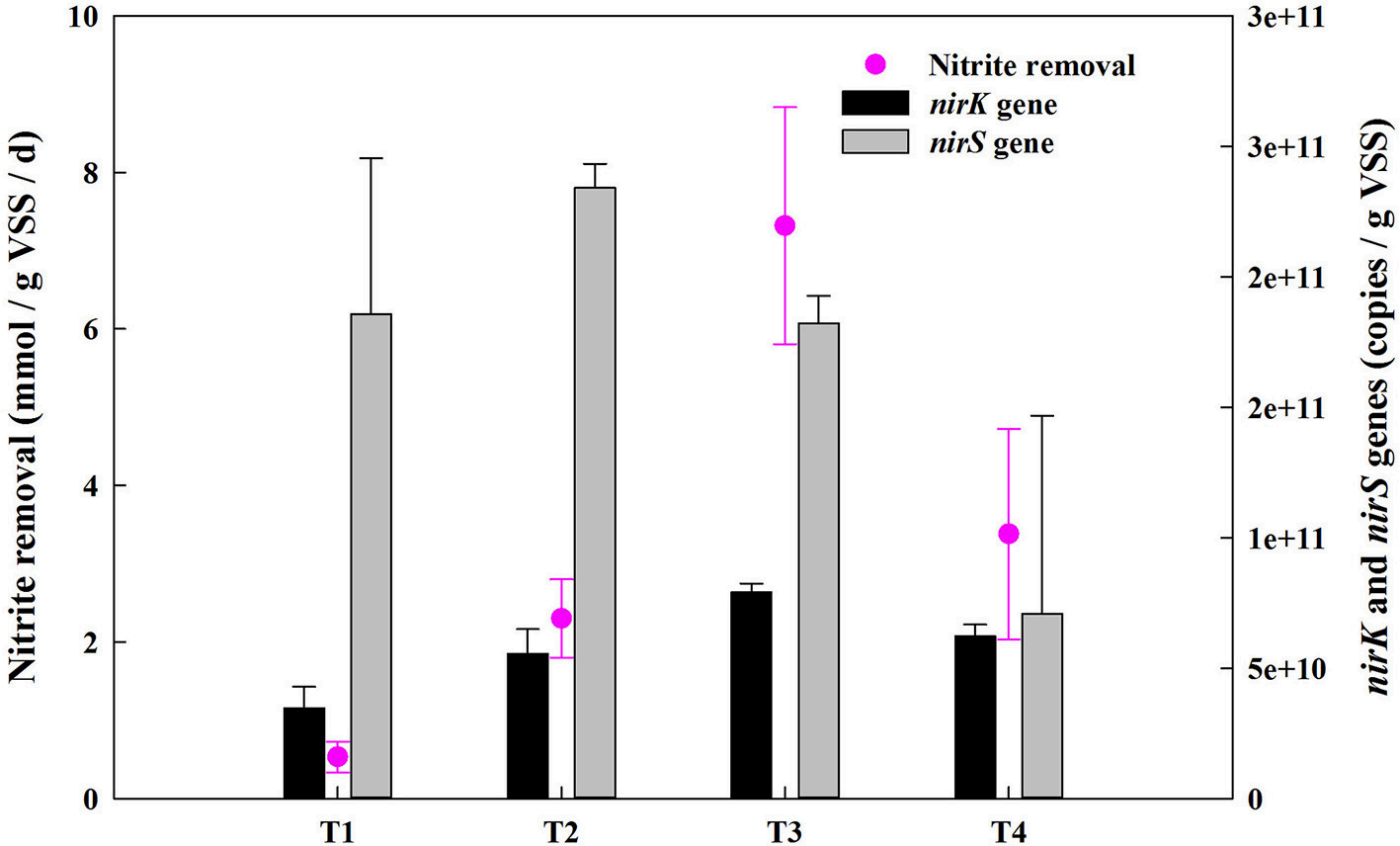
Nitrite removal of the consortia and the abundance of functional genes at different O_2_:CH_4_ ratios in aerobic methane oxidation coupled with denitrification (AME-D) process. T1, Treatment 1 (0% O_2_ and 100% CH_4_); T2, Treatment 2 (5% O_2_ and 95% CH_4_); T3, Treatment 3 (20% O_2_ and 80% CH_4_); T4, Treatment 4 (50% O_2_ and 50% CH_4_).

The concentrations of ${\text{NO}}_{2}^{-}$-N, ${\text{NH}}_{4}^{+}$-N and ${\text{NO}}_{3}^{-}$-N were measured at the beginning and the end of incubation. Results showed that concentrations of ${\text{NH}}_{4}^{+}$-N and ${\text{NO}}_{3}^{-}$-N decreased slightly by the end of the experiment and were detected in all final samples (Supplementary Table 2). There was a jump in the nitrite removal from 0.53 mmol ${\text{NO}}_{2}^{-}$-N/gVSS/d to 7.32 mmol ${\text{NO}}_{2}^{-}$-N/gVSS/d when the O_2_:CH_4_ ratio was increased from 0 to 0.25 (Figure 4). However, the nitrite removal decreased by 53.8% as the O_2_:CH_4_ ratio was increased from 0.25 to 1 (*p* < 0.05). The amount of reduced nitrogen released as NO was very low (0.13–0.32 μM; Table 2). In addition, the percentage of the reduced nitrogen emitted as N_2_O decreased from 37.96 to 12.30% when the O_2_:CH_4_ ratio increased from 0 to 0.25 (Table 2). This percentage at the O_2_:CH_4_ ratio of 1 was about 2 times higher than that at the O_2_:CH_4_ ratio of 0.25, while this difference was insignificant (*p* > 0.05; Table 2). The highest nitrite removal and the low percentage of NO-N and N_2_O-N in total reduced nitrogen were observed at the O_2_:CH_4_ ratio of 0.25. Based on the results, the O_2_:CH_4_ ratio of 0.25 was proposed as the optimal ratio for this AME-D system.

**Table 2:** Reduction of ${\text{NO}}_{2}^{-}$-N and accumulation of NO-N and N_2_O-N at different O_2_:CH_4_ ratios in aerobic methane oxidation coupled with denitrification (AME-D) process.

**O_2_/CH_4_ ratio**	**0^a^**	**0.05^b^**	**0.25^c^**	**1^d^**
Reduced ${\text{NO}}_{2}^{-}$-N (μmol)	5.06 ± 1.88	21.87 ± 4.74	69.54 ± 14.37	32.11 ± 12.78
Accumulated NO-N (μmol)	0.18 ± 0.03	0.24 ± 0.03	0.14 ± 0.03	0.32 ± 0.03
Accumulated N_2_O-N (μmol)	1.82 ± 0.27	3.58 ± 0.23	8.24 ± 0.71	7.17 ± 0.47
Accumulated NO-N/ Reduced ${\text{NO}}_{2}^{-}$-N	3.96 ± 1.50%	1.14 ± 0.18%	0.20 ± 0.03%	1.17 ± 0.68%
Accumulated N_2_O-N/ Reduced ${\text{NO}}_{2}^{-}$-N	37.96 ± 9.13%	16.78 ± 3.06%	12.30 ± 3.27%	26.11 ± 14.34%

a *(0% O*_2_*; 100% CH_4_)*.

b *(5% O*_2_*; 95% CH_4_)*.

c *(20% O*_2_*; 80% CH_4_)*.

d *(50% O*_2_*; 50% CH_4_)*.

**Figure 5:**
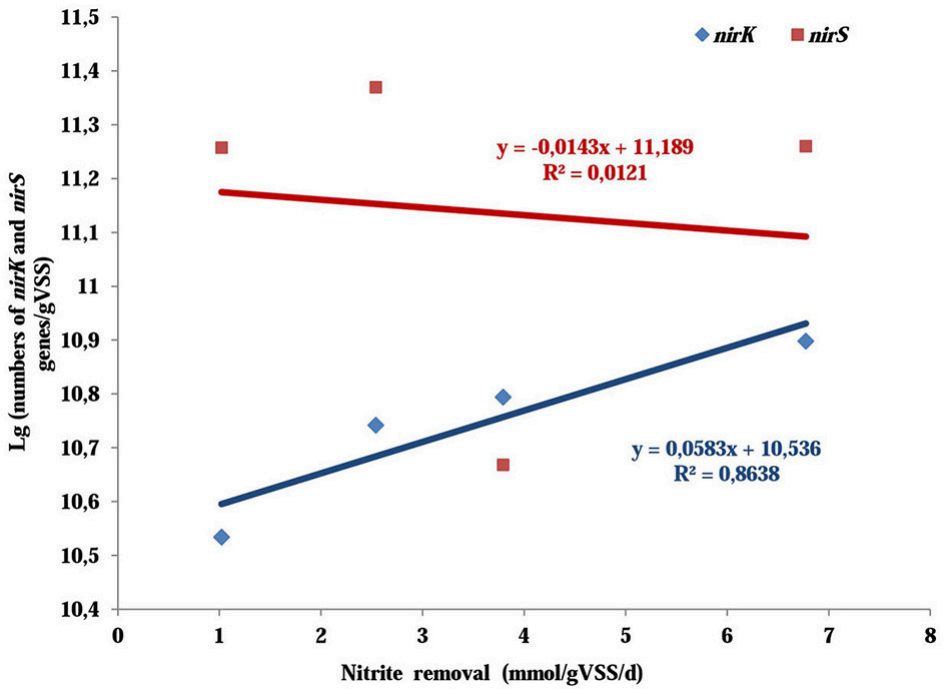
The relationships between nitrite removal and the abundance of *nirK*/*nirS* genes in aerobic methane oxidation coupled with denitrification (AME-D) process. Linear regressions were used to test the correlation between nitrite removal and the abundance of *nirK*/*nirS* genes.

Nitrite reduction is catalyzed by nitrite reductase which are found in two different forms: copper nitrite reductase encoded by *nirK* gene and cytochrome cd_1_-containing nitrite reductase encoded by *nirS* gene (Wang et al., 2014). Investigating the difference in *nirK* and *nirS* gene abundance might provide further evidences for the variation of nitrite removal. The abundance of *nirK* gene was more than doubled as the O_2_:CH_4_ ratio was increased from 0 to 0.25 (from 3.47 × 10^10^ copies/gVSS up to 7.91 × 10^10^ copies/gVSS). However, it descended to 6.23 × 10^10^ copies/gVSS as the O_2_:CH_4_ ratio was further raised to 1. In contrast, copy numbers of *nirS* gene displayed no significant change (a slight increase from 1.82 copies/gVSS to 2.34 × 10^11^ copies/gVSS) when O_2_:CH_4_ ratio was increased from 0 to 0.25. However, it went down significantly to 7.10 × 10^10^ copies/gVSS at higher O_2_:CH_4_ ratio of 1. In addition, the linear correlation analysis revealed that *nirK* gene copies were positively correlated with nitrite removal (*r*^2^ = 0.8639, *p* = 0.0707), whereas *nirS* gene copies had a slightly negative correlation with the nitrite removal (*r*^2^ = 0.0121, *p* = 0.8899; Figure 5). If the nitrite removal was considered as the representation of the denitrifying conditions in the corresponding treatment, the higher nitrite removal indicated the better denitrifying conditions in this treatment. Thereby, the linear coorelation analyses suggest that *nirK*-type denitrifiers might be more responsive to the denitrifying conditions than *nirS*-type denitrifiers (Yoshida et al., 2010).

## Discussion

### The optimal O_2_:CH_4_ ratio with highest nitrite removal of AME-D process

The results from the batch experiment showed that the peak nitrite removal (7.32 mmol ${\text{NO}}_{2}^{-}$-N/gVSS/d) was obtained at an O_2_:CH_4_ ratio of 0.25. As ${\text{NH}}_{4}^{+}$-N and ${\text{NO}}_{3}^{-}$-N are the preferred microbial nitrogen sources (Modin et al., 2007), their presence throughout the incubation period (Supplementary Table 2) indicated that nitrite was negligibly consumed as a nitrogen source for assimilation. Therefore, nitrite removal from the media was through dissimilatory, i.e., denitrification. Additionally, the low percentage of the consumed ${\text{NO}}_{2}^{-}$-N emitted as N_2_O-N (12.30%) and NO-N (0.20%, Table 2) at the O_2_:CH_4_ ratio of 0.25 indicated that enough carbon sources provided by the methanotrophs may allow denitrifiers to reduce almost 87.5% of ${\text{NO}}_{2}^{-}$-N through complete denitrification. At a lower O_2_:CH_4_ ratio of 0.25, it was consistent with our hypothesis that nitrite removal were not inhibited by O_2_, and were stimulated by the increased carbon provided by methanotrophic metabolism. However, nitrite removal was less effective at higher O_2_:CH_4_ ratios with higher oxygen concentrations, which was corroborated by the lower abundance of denitrifying genes (Figure 4).

The conclusion that nitrite removal was stimulated by carbon sources released from aerobic methane oxidation at a O_2_:CH_4_ ratio lower than or equal to 0.25 could be supported by the variation of intermediate concentrations among four treatments. Concentrations of accumulated intermediates were higher and higher in the bulk media with the O_2_:CH_4_ ratios increasing from 0 to 0.25 (Figure 3). It may be attributed to the increased O_2_ concentration in these treatments. Morinaga et al. (1979) and Costa et al. (2001) demonstrated that the O_2_ level would significantly impact the consumption and production of metabolites in methane metabolism. However, the effect was not unambiguously determined. Morinaga et al. (1979) observed formaldehyde accumulation under oxygen-limited conditions, whereas Costa et al. (2001) discovered that formaldehyde accumulated under oxygen-excessive conditions. Our results indicated that the increased oxygen level promoted intermediates accumulation in methane metabolism. Low level of available carbon sources for denitrifiers in Treatment 1 resulted in the lowest nitrite removal. Once O_2_ was largely induced in the headspace in Treatment 2 and 3, concentrations of intermediates increased in the liquid bulk. The observation of simultaneous higher nitrite removal and lower ratio of accumulated N_2_O-N:consumed ${\text{NO}}_{2}^{-}$-N indicated that complete denitrifying activity was improved by these accumulated intermediates.

**Figure 6:**
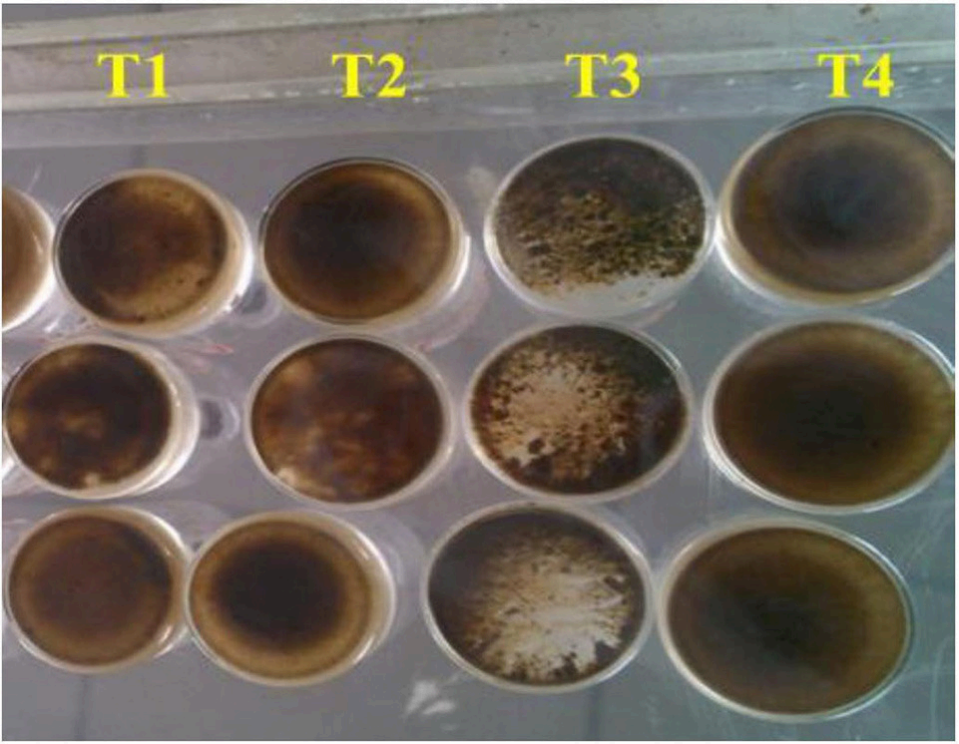
Configuration of activated sludge in four treatments at the end of incubation during aerobic methane oxidation coupled with denitrification (AME-D) process. The phenomenon of aggregation is apparent in treatment 3 (T3) while activated sludge in other treatments were dispersive. T1, Treatment 1 (0% O_2_ and 100% CH_4_); T2, Treatment 2 (5% O_2_ and 95% CH_4_); T3, Treatment 3 (20% O_2_ and 80% CH_4_); T4, Treatment 4 (50% O_2_ and 50% CH_4_).

The ability of denitrifiers to resist the inhibition of O_2_ at the optimal O_2_:CH_4_ ratio may be due to two reasons. Firstly, the abundance of *nirK*-harboring denitrifiers was the highest among the four treatments and these denitrifiers are able to tolerate higher O_2_ levels (Desnues et al., 2007). Secondly, the sludge aggregation/granulation in anoxic micro-environments can lessen the O_2_ exposure of the denitrifiers. As shown in Figure 6, at the O_2_:CH_4_ ratio of 0.25, suspended biomass formed granule-like agglomerates with an average diameter of about 2 mm, while such effect did not occur for the O_2_:CH_4_ ratios of 0, 0.05, and 1. Sludge aggregation has also been observed in an AME-D system with an optimized O_2_ level that had the highest nitrogen removal rate (Thalasso et al., 1997). It can be therefore concluded that sludge aggregation at an optimal O_2_:CH_4_ ratio could improve nitrite removal. The spatial distribution of microorganisms and O_2_ level within the aggregates should be investigated in the future.

With regard to the effect of the O_2_:CH_4_ ratio on denitrification, Sun et al. (2013) observed a similar phenomenon that the O_2_:CH_4_ ratio affected nitrogen removal of AME-D process in a membrane biofilm bioreactor (MBfR). Their optimal O_2_:CH_4_ ratio of 1.5 was considerably higher than the one in this study. It is likely that the spatial arrangement of the microbial community within a well-developed biofilm would allow for greater tolerance to O_2_, as compared to a suspended culture. They speculated that greater metabolite excretion by the methanotrophs improved nitrate removal performance initially, and that excessive O_2_ caused the significant drop in the denitrifying rate when O_2_:CH_4_ ratio increased to 2.0. The results from this study corroborate the trend that Sun et al. (2013) observed.

Methane oxidation activity dramatically decreased when O_2_:CH_4_ ratio increased from 0 to 1 (Figure 2). It was probably due to the large change in methane availability. Li et al. (2014) observed a similar trend with methanotrophic activity of landfill cover soils. The methane oxidation activity at O_2_:CH_4_ ratio of 0.25 (4 × 10^4^ ppmv O_2_/2 × 10^5^ ppmv CH_4_) was almost 4-fold higher than that at O_2_:CH_4_ ratio of 4.00 (2 × 10^5^ ppmv O_2_/5 × 10^4^ ppmv CH_4_) (Supplementary Table 3). In their research, methane oxidation activity was much more sensitive to CH_4_ than O_2_, and a drop of CH_4_ concentration would result in a simultaneous decrease of methane oxidation activity. Additionally, copy numbers of the *pmoA* gene decreased as the methane concentration decreased (Baani and Liesack, 2008; Li et al., 2014). In the current research, CH_4_ concentration declined from 100 to 50% and O_2_ concentrations rose from 0 to 50% for the ratios tested. This suggests that a decrease in methane oxidation activity with a substantial increase in O_2_:CH_4_ ratio is plausible. However, it is unexpected that the high *pmoA* gene copy numbers and methane oxidation activity were observed in Treatment 1 in the absence of O_2_. Rechecking the gaseous composition of the headspace in this treatment at the beginning of the incubation, it was discovered that trace amount (0.012%) of O_2_ can still be detected after the sludge was sparged with pure CH_4_ (99.99%). This trace level of O_2_ may result in the observed CH_4_ consumption. However, further investigations are still required to focus on examining if anaerobic methane oxidation contributed to CH_4_ consumption when this trace amount of O_2_ was depleted.

### Thermodynamic speculation for metabolic pathways of AME-D process

Acetate, citrate, and formaldehyde were detected as three primary compounds detected during the AME-D process, while methanol, formate, and butyrate occurred in trace quantities. It is difficult to postulate which were the main substrates for the denitrifiers as their levels of consumption relative to production were not known. However, thermodynamic analysis of AME-D process may provide useful information for speculating the actual metabolic pathway (all of the following thermodynamic analyses are based on aerobic methane oxidation coupled with complete denitrification).

Methanol is considered as the most effective intermediate for denitrification according to the review of the AME-D process (Zhu et al., 2016). The reactions (Equations 3–5) related to energy production contained in AME-D process using ${\text{NO}}_{2}^{-}$ as the denitrifying electron acceptor were shown in Table 3. These equations are based on one electron equivalent (eeq). Assuming that at least *X* (<1) mol of methanol is needed by aerobic methanotrophs for their requirement of cell synthesis and maintenance when one mole of CH_4_ is oxidized to methanol, the remaining part of methanol can be used for denitrification as the electron donor. An energy-balanced equation for the AME-D process can be described as Equation (6).

**Table 3:** Stoichiometric equations in aerobic methane oxidation coupled with denitrification (AME-D) process based on theoretical hypotheses.

**Equation**	**Stoichiometric equation**	**ΔG^0′^**
(3)	$\frac{1}{2}{\text{CH}}_{4}+\frac{1}{4}{\text{O}}_{2}=\frac{1}{2}{\text{CH}}_{3}\text{OH}$	−62.34 kJ/eeq
(4)	$\frac{1}{6}{\text{CH}}_{3}\text{OH}+\frac{1}{4}{\text{O}}_{2}=\frac{1}{6}{\text{CO}}_{2}+\frac{1}{3}{\text{H}}_{2}\text{O}$	−115.56 kJ/eeq
(5)	$\frac{1}{6}\text{C}{\text{H}}_{3}\text{OH}+\frac{1}{3}{\text{NO}}_{2}^{-}+\frac{1}{3}{\text{H}}^{+}=\frac{1}{6}{\text{N}}_{2}+\frac{1}{6}\text{C}{\text{O}}_{2}+\frac{1}{2}{\text{H}}_{2}\text{O}$	−129.40 kJ/eeq

(6) $$6\cdot \;\Delta G_{Eq.\left(4\right)}\cdot \varepsilon \cdot X+2\cdot \left[\Delta G_{Eq.\left(3\right)}-0.5\cdot \Delta G_{xy}\right]+\Delta G_{m}=0$$

Where ε, is the energy transfer efficiency a value of 0.37 (McCarty, 2007). [Δ*G*_*Eq*.(3)_ – 0.5Δ*G*_*xy*_] denotes the net energy production for the oxidation from CH_4_ to methanol after considering the energy input for the mono-oxygenase and the required reducing equivalent, which equals to 47.26 kJ/eeq. Δ*G*_*m*_ represents the minimum maintenance energy requirement with a value of about 8.1 kJ/mol oxidized CH_4_ (Modin et al., 2007). The exhaustive description of calculation processes for [Δ*G*_*Eq*.(3)_ – 0.5Δ*G*_*xy*_] and Δ*G*_*m*_ is presented by Zhu et al. (2016). From Equation (6), the value derived for *X* is 0.40. Because the energy required by cell synthesis has not been considered during the above calculation, the theoretical maximum quantity of methanol used by denitrifiers was 0.60 mol. This means that the maximum proportion of methanol that can be captured by denitrifiers is 60%. The overall reaction of AME-D process in the presence of ${\text{NO}}_{2}^{-}$ was described as Equation (7), a combination of the proposed value and Equations (3–5).

(7) $$\begin{array}{l} {\text{CH}}_{4}+\frac{11}{10}{\text{O}}_{2}+\frac{6}{5}{\text{NO}}_{2}^{-}+\frac{6}{5}{\text{H}}^{+}=\frac{3}{5}{\text{N}}_{2}+{\text{CO}}_{2}+\frac{13}{5}{\text{H}}_{2}\text{O}\\ \;\Delta {\text{G}}^{0^{\prime }}=-867.86\text{kJ}/\text{mol }{\text{CH}}_{4} \end{array}$$

All six substrates detected in this study were potential carbon sources for denitrifiers. In order to understand which substrates were likely the functional intermediates, thermodynamic analysis was performed based on the chemical data associated with the O_2_:CH_4_ ratio of 0.25. During the process of thermodynamic derivation, all of organics detected in the bulk liquid were individually chosen as the trophic link of aerobic methane oxidation and denitrification. After several iterations, the same general equation (Equation 8) for the AME-D process, which was in agreement with CH_4_ and ${\text{NO}}_{2}^{-}$ consumption was obtained through three different approaches:

1. 4.34% of consumed CH_4_-C flowed to denitrifiers using methanol as the trophic link;
2. 5.22% of consumed CH_4_-C flowed to denitrifiers using butyrate as the trophic link;
3. 6.52% of consumed CH_4_-C flowed to denitrifiers using formaldehyde as the trophic link. This means that methanol, butyrate and formaldehyde released by aerobic methanotrophs were three possible intermediates that could be used as carbon sources by denitrifiers at an O_2_:CH_4_ ratio of 0.25. However, acetate could not be an active carbon source under this condition in our study, although it was a feasible electron donor with highest denitrifying potential (Hallin and Pell, 1998). This conclusion is further supported by the increased copy numbers of *nirK* genes, which would have decreased if acetate was the main active electron donor for denitrifiers (Li et al., 2015).

(8) $$\begin{array}{l} {\text{CH}}_{4}+\frac{89}{46}{\text{O}}_{2}+\frac{2}{23}{\text{NO}}_{2}^{-}+\frac{2}{23}{\text{H}}^{+}=\frac{1}{23}{\text{N}}_{2}+{\text{CO}}_{2}+\frac{47}{23}{\text{H}}_{2}\text{O}\\ \;\Delta {\text{G}}^{0^{\prime }}=-821.65\text{kJ}/\text{mol}{\text{CH}}_{4} \end{array}$$

Based on the above analysis, it was evident that the percentage of carbon flow from methane to denitrification (4.34–6.52%) was much lower than the ideal flow (60%). Further improvement in the carbon flow is vital to enhance the AME-D denitrification rates.

### Implication of O_2_:CH_4_ ratio control for nitrogen removal in AME-D process

The effect of the O_2_:CH_4_ ratio was demonstrated to significantly impact nitrogen removal during the AME-D process through contribution of the carbon metabolites generated by the methanotrophs and oxygen inhibition. To date, most studies have considered only the individual impact of O_2_ on the apparent nitrogen removal rate of the AME-D process (Werner and Kayser, 1991; Thalasso et al., 1997; Modin et al., 2010), whereas the combined effect of O_2_ and CH_4_ and associated mechanisms are rarely investigated. It is necessary to address the impact of O_2_:CH_4_ ratio on CH_4_ and ${\text{NO}}_{3}^{-}$/${\text{NO}}_{2}^{-}$ metabolism in the AME-D process, rather than the tendency of methane oxidation rates and denitrifying activities under different gaseous environments. The knowledge will allow a better understanding of the specific roles of the O_2_:CH_4_ ratio in CH_4_ and ${\text{NO}}_{3}^{-}$/${\text{NO}}_{2}^{-}$ metabolism. It is expected that this will contribute to well-founded strategies that will improve nitrogen removal, one of bottle-necks in the application of the AME-D process.

In this study, the optimal O_2_:CH_4_ ratio for denitrification was found to be 0.25. At this point, denitrifying activity reached the highest level of 7.32 mmol ${\text{NO}}_{2}^{-}$-N/gVSS/d. When the O_2_:CH_4_ ratio was below the optimal ratio, nitrite removal was improved with the increased O_2_:CH_4_ ratio, presumably due to an increase in available substrates released by aerobic methanotrophs. Methanol, butyrate and formaldehyde were thermodynamically speculated as the main active intermediates of the AME-D process. When the O_2_:CH_4_ ratio was above the optimal ratio, nitrite removal was presumably inhibited by the excessive O_2_. These results indicate that adjusting the O_2_:CH_4_ ratio can improve the cooperation between aerobic methanotrophs and denitrifiers to obtain better nitrogen removal performance using the AME-D process.

## Author contributions

JZ: contributed to the conception, experimental design, acquisition, analysis, and interpretation of data, and article drafting; XX, MY: analyzed and interpreted data; HW, ZM: contributed to data acquisition; WW: supervised the student and revised the article.

### Conflict of interest statement

The authors declare that the research was conducted in the absence of any commercial or financial relationships that could be construed as a potential conflict of interest.
