# Supplementary material for: Optimum O2:CH4 Ratio Promotes the Synergy between Aerobic Methanotrophs and Denitrifiers to Enhance Nitrogen Removal
Source: Front Microbiol. 2017 Jun 16;8:1112. doi: 10.3389/fmicb.2017.01112 (PMC5472701; doi:10.3389/fmicb.2017.01112)
Supplement: Supplementary file 1 [file Table1.PDF]

Supplementary Table 1. Primers and thermal cycling conditions for qPCR

Target genes	Primers	Sequence(5'-3')	Amplicon size(bp)	Thermal conditions	Reference
***pmo*A**	A189f<br>mb661	GGN GAC TGG GAC TTC TGG<br>CCG GMG CAA CGT CYT TAC C	510	95°C, 3 min, 1cycle<br>95°C for 15 s, 60 °C for 30 s, 72°C for 30 s, 37 cycles	(Liu et al., 2014)
nirK	F1aCu<br>R3Cu	ATC ATG GT(C/G) CTG CCG CG<br>GCC TCG ATC AG(A/G) TTG TGG TT	473	95°C, 3 min, 1cycle<br>95°C for 10 s, 58 °C for 30 s, 72°C for 30 s, 35 cycles	(Throback et al., 2004)
nirS	cd3aF<br>R3cd	GT(C/G) AAC GT(C/G) AAG GA(A/G) AC(C/G) GG<br>GA(C/G) TTC GG(A/G) TG(C/G) GTC TTG A	425	95°C, 3 min, 1cycle<br>95°C for 10 s, 57 °C for 30 s, 72°C for 30 s, 35 cycles	(Throback et al., 2004)

Liu, J.J., Sun, F.Q., Wang, L., Ju, X., Wu, W.X., and Chen, Y.X. (2014). Molecular characterization of a microbial consortium involved in methane oxidation coupled to denitrification under micro-aerobic conditions. *Microbial Biotechnol.* 7, 64-76.

Throback, I.N., Enwall, K., Jarvis, A., and Hallin, S. (2004). Reassessing PCR primers targeting *nirS*, *nirK* and *nosZ* genes for community surveys of denitrifying bacteria with DGGE. *FEMS Microbiol. Ecol.* 49, 401-417.
