# Supplementary material for: Optimum O2:CH4 Ratio Promotes the Synergy between Aerobic Methanotrophs and Denitrifiers to Enhance Nitrogen Removal
Source: Front Microbiol. 2017 Jun 16;8:1112. doi: 10.3389/fmicb.2017.01112 (PMC5472701; doi:10.3389/fmicb.2017.01112)
Supplement: Supplementary file 2 [file Table2.PDF]

Supplementary Table 2. Concentrations of NH_4^+-N, NO_3^--N and NO_2^--N in the medium of each treatment at the beginning and the end of incubation during aerobic methane oxidation coupled with denitrification (AME-D) process

Treatments	Beginning of incubation [a]			End of incubation		
	NH_4^+-N (mg L^{-1})	NO_3^--N (mg L^{-1})	NO_2^--N (mg L^{-1})	NH_4^+-N (mg L^{-1})	NO_3^--N (mg L^{-1})	NO_2^--N (mg L^{-1})
**T 1**	0.070±0.008	3.82±0.44	76.19±1.59	0.015±0.001	3.89±0.22	72.84±0.88
**Control 1**				0.073	4.48	75.20
**T 2**				0.015±0.001	3.76±0.20	67.41±2.21
**Control 2**				0.073	4.48	77.62
**T 3**				0.011±0.001	2.39±0.06	45.73±6.71
**Control 3**				0.070	4.56	78.18
**T 4**				0.017±0.001	3.47±0.10	58.35±5.96
**Control 4**				0.070	4.68	73.34

[a]: The initial concentrations of NH_4^+-N, NO_3^--N and NO_2^--N in 4 treatments were identical.
