# Supplementary material for: Optimum O2:CH4 Ratio Promotes the Synergy between Aerobic Methanotrophs and Denitrifiers to Enhance Nitrogen Removal
Source: Front Microbiol. 2017 Jun 16;8:1112. doi: 10.3389/fmicb.2017.01112 (PMC5472701; doi:10.3389/fmicb.2017.01112)
Supplement: Supplementary file 3 [file Table3.pdf]

Supplementary Table 3.

Estimated cell-specific CH_4 oxidation rates of methanotrophic bacteria ($\times 10^{-16}$ mol cell^{-1} h^{-1}) (Li et al., 2014)

**Methane oxidation rate ($\times 10^{-16}$ mol cell^{-1} h^{-1})**		**CH_4 (ppmv)**		
		5×10^4	1×10^5	2×10^5
**O_2 (ppmv)**	5×10^4	14.1	33.1	69.9 (O_2:CH_4=0.25)
	1×10^5	15.0	34.2 (O_2:CH_4=1)	74.3
	2×10^5	18.1 (O_2:CH_4=4)	38.1	76.6

Li, H., Chi, Z., Lu, W., and Wang, H. (2014). Sensitivity of methanotrophic community structure, abundance, and gene expression to CH_4 and O_2 in simulated landfill biocover soil. *Environ Pollut* 184, 347-353.
