# Supplementary material for: Optimum O2:CH4 Ratio Promotes the Synergy between Aerobic Methanotrophs and Denitrifiers to Enhance Nitrogen Removal
Source: Front Microbiol. 2017 Jun 16;8:1112. doi: 10.3389/fmicb.2017.01112 (PMC5472701; doi:10.3389/fmicb.2017.01112)
Supplement: Supplementary file 4 [file Image1.pdf]

Supplementary Material

Optimum O_2:CH_4 ratio promotes the synergy between aerobic methanotrophs and denitrifiers to enhance nitrogen removal

Jing Zhu, Xingkun Xu, Mengdong Yuan, Hanghang Wu, Zhuang Ma, Weixiang Wu*

*** Correspondence: Weixiang Wu**: weixiang@zju.edu.cn

1 Supplementary Figures and Tables

1.1 Supplementary Figures

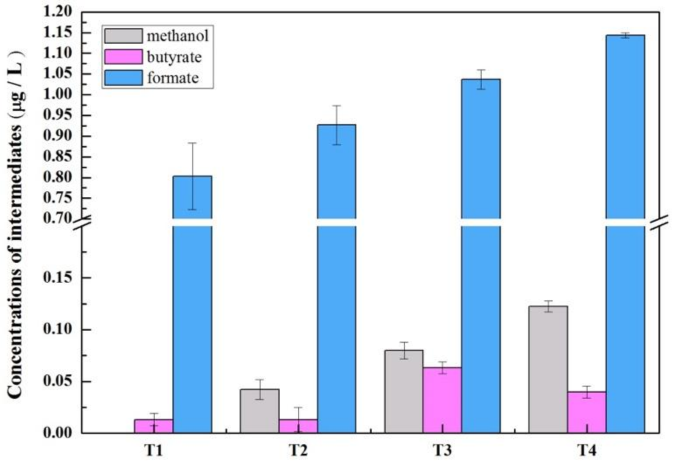

Supplementary Figure 1. Accumulation of methanol, butyrate, and formate in the liquid at different O_2:CH_4 ratios in aerobic methane oxidation coupled with denitrification (AME-D) process. T1, Treatment 1 (0% O_2 and 100% CH_4); T2, Treatment 2 (5% O_2 and 95% CH_4); T3, Treatment 3 (20% O_2 and 80% CH_4); T4, Treatment 4 (50% O_2 and 50% CH_4).
